# Pathological Evaluation of Diffuse Gliomas Using IDH1 and ATRX in a Resource-Limited Setting

**DOI:** 10.7759/cureus.65551

**Published:** 2024-07-27

**Authors:** Ramya Chitturi, Aparna Chinnam

**Affiliations:** 1 Pathology, Guntur Medical College, Guntur, IND

**Keywords:** immunohistochemistry (ihc), cns tumors, atrx, idh1, diffuse glioma

## Abstract

Introduction: Classification of gliomas based on tumor histology remains the gold standard in the diagnosis and prognosis of gliomas. However, the recent World Health Organization (WHO) classification has included molecular studies for diagnosis and prognostication. Immunohistochemical markers such as isocitrate dehydrogenase 1 (IDH1) and alpha thalassemia/mental retardation syndrome X-linked (ATRX) can be used for the diagnosis and prognosis of the majority of gliomas.

Objectives: We aim to study the frequencies of IDH1 and ATRX mutations in diffuse gliomas using surrogate immunohistochemical markers and correlate histopathological findings of gliomas with immunohistochemical findings.

Material and methods: This was a retrospective study of one-year duration from January 2022 to December 2022, conducted in the department of pathology. Relevant data was retrieved from medical records. Histopathology blocks were collected and sent for immunohistochemical studies using tissue microarray for IDH1 and ATRX.

Statistical analysis: Qualitative data were expressed in percentages and proportions. The difference in proportion was calculated using the chi-square test, and a p-value of <0.005 was taken as significant.

Results: A total of 51 cases of diffuse gliomas were included in the study. The frequency of IDH1-positive diffuse astrocytomas was 33 (64.7%), and loss of ATRX was seen in 12 (23.5%) cases.

Conclusion: Immunohistochemistry serves as a surrogate marker to detect molecular alterations in diffuse gliomas.

## Introduction

Traditionally, the classification of central nervous system (CNS) tumors was based extensively on histological features, but the new World Health Organization (WHO) classification of CNS tumors introduced molecular markers that provide diagnostic and prognostic information. The WHO also emphasizes the importance of integrated diagnosis combining morphology and molecular studies along with layered reporting [1]. Even in developed countries, molecular studies are not readily available in all centers, and although they are available, they need expertise to interpret, and they are expensive. In developing countries such as India, molecular studies are available only in a few selected places, and they are not affordable to common people. Since many of these genetic parameters can be assessed using surrogate immunohistochemical (IHC) markers, IHC offers an easily available, affordable test to detect molecular alterations in tumors [2].

Routine IHC panels for diffuse astrocytomas include glial fibrillary acidic protein (GFAP), isocitrate dehydrogenase 1 (IDH1) and alpha thalassemia/mental retardation syndrome X-linked (ATRX), p53, and Ki67. There are various studies using different IHC markers for the stratification of glial tumors, but the two most common markers that form baseline investigations are IDH1 and ATRX. Chatterjee et al. [3] have proved that combined IDH1 and ATRX IHC can accurately confirm the molecular nature of most grade 1 astrocytomas and grade 3 astrocytoma cases, thereby avoiding the need for expensive investigations such as gene sequencing. The evaluation of IDH1 and ATRX status in gliomas has both diagnostic and prognostic value as it helps in differentiating gliomas from reactive gliosis, primary glioblastoma (GB) from secondary glioblastoma, and pilocytic astrocytoma (PA) WHO grade 1 from grade 2 astrocytoma [4].

Various methods of detecting IDH mutations are present, such as DNA sequencing and pyrosequencing, but all these procedures are time-consuming, laborious, and not available in all centers [5]. Immunohistochemistry acts as a surrogate marker for these genetic alterations. It is the most sensitive and specific test for the detection of IDH1 R132H mutation [5]. IDH2 mutation is extremely rare in astrocytic tumors and is more often associated with an oligodendroglial phenotype. They can only be detected by gene sequencing. Hartmann et al. [6] found IDH2 mutation in 0.9%, 0.9%, 4.7%, and 5.2% in grade 2 astrocytoma, grade 3 astrocytoma, grade 2 oligodendroglioma, and grade 3 oligodendroglioma, respectively. So, IDH2 mutation testing is not done routinely.

The purpose of the present study is to evaluate the frequency of IDH1 and ATRX mutations in diffuse gliomas and correlate them with histopathological findings.

## Materials and methods

This was a retrospective observational study conducted in the department of pathology. Relevant data such as age, sex, location of the tumor, and clinical and radiological details were retrieved from the archives. The inclusion criteria were all cases of diffuse gliomas. The exclusion criteria were pediatric gliomas and CNS tumors other than gliomas. Institutional ethics committee clearance was taken.

Formalin-fixed paraffin blocks were used for tissue microarray (TMA) construction. All the paraffin blocks were marked for the best representative area with the help of corresponding hematoxylin and eosin (H&E)-stained slides. Sections were deparaffinized through xylene and then rehydrated. Slides were then incubated in ready-to-use Peroxidazed 1 by Biocare Medical (Pacheco, CA) for five minutes to block endogenous peroxidase activity. Antigen retrieval was performed using a Decloaking Chamber (Biocare Medical), and incubation with primary antibodies was done at 40°C. The antibodies used were IDH1 R132H (Clone IHC 132, RTU) and ATRX (Clone ZR10, Dilution 1:100) from Biocare Medical. MACH 1 Universal Polymer Detection system (Biocare Medical) was used, and the chromogenic substrate was Betazoid Diaminobenzidine (DAB) (Biocare Medical). The sections were washed and counterstained with hematoxylin. A core was considered suitable for evaluation if at least 50% of the tumor cells were retained in the core. Positive internal control for IDH1 and ATRX was a normal brain, which was IDH1 wild type (negative) and showed retained expression (positive) for ATRX.

Frequencies of various grades of astrocytic and oligodendroglial tumors were assessed by histomorphology. The presence of IDH1 and ATRX mutations in different grades of astrocytic and oligodendroglial tumors was studied. The findings were analyzed using Microsoft Excel Worksheet (Microsoft Corp., Redmond, WA) and the Statistical Package for the Social Sciences (SPSS) version 20.0 for Windows (IBM SPSS Statistics, Armonk, NY). Qualitative data were expressed in the form of percentages and proportions. The difference in proportion was calculated using the chi-square test, and a p-value of <0.005 was taken as significant.

## Results

There were 51 cases of gliomas. The age of the patients ranged from 20 years to 80 years, with the mean age being 47.65 years. The male-to-female ratio was 1.8:1. The clinical presentation ranged from headache to seizures and hemiplegia. Twenty-eight gliomas were right-sided, 20 were left-sided, and one each was located at the fourth ventricle, optic nerve, and craniovertebral junction. The histological types included in the present study were 19 cases of grade 2 astrocytomas, nine cases of grade 3 astrocytomas, nine cases of grade 4 astrocytoma, seven cases of glioblastomas (GB), five cases of grade 2 oligodendroglioma, and two cases of grade 3 oligodendroglioma. The most common tumor in the present study was grade 2 astrocytoma (37.2%). IDH1 mutant (positive) tumors were seen in a total of 33 (64.7%) cases of diffuse gliomas (Table 1).

**Table 1:** Frequencies of IDH1 and ATRX mutations in the present study IDH1: isocitrate dehydrogenase 1, ATRX: alpha thalassemia/mental retardation syndrome X-linked

Type of gliomas	IDH1 mutant	IDH1 negative	ATRX retained	ATRX negative
Grade 2 astrocytoma (19)	11 (57.9%)	8 (42.1%)	15 (78.9%)	4 (21.1%)
Grade 3 astrocytoma (9)	6 (66.7%)	3 (33.3%)	6 (66.7%)	3 (33.3%)
Grade 4 astrocytoma (9)	9 (100%)	0 (0%)	4 (44.4%)	5 (55.6%)
Glioblastoma (7)	0 (0%)	7 (100%)	7 (100%)	0 (0%)
Grade 2 oligodendroglioma (5)	5 (100%)	0 (0%)	5 (100%)	0 (0%)
Grade 3 oligodendroglioma (2)	2 (100%)	0 (0%)	2 (100%)	0 (0%)

**Figure 1:**
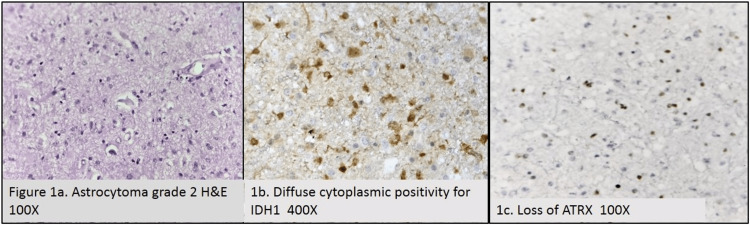
Grade 2 astrocytoma (H&E, 100×) (1a), diffuse cytoplasmic positivity for IDH1 (400×) (1b), and loss of ATRX (100×) (1c) H&E: hematoxylin and eosin, IDH1: isocitrate dehydrogenase 1, ATRX: alpha thalassemia/mental retardation syndrome X-linked

**Figure 2:**
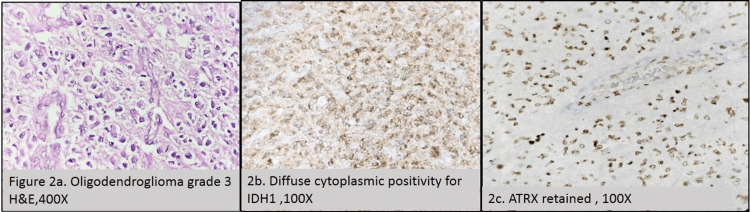
Grade 3 oligodendroglioma (H&E, 400×) (2a), diffuse cytoplasmic positivity for IDH1 (100×) (2b), and ATRX retained (100×) (2c) H&E: hematoxylin and eosin, IDH1: isocitrate dehydrogenase 1, ATRX: alpha thalassemia/mental retardation syndrome X-linked

In grade 2 astrocytoma, tumor cells showed increased cellularity with mild nuclear atypia. The glial cells showed abundant cytoplasm with diffuse infiltration into the brain parenchyma. Out of 19 cases of grade 2 astrocytomas, 57.9% of cases showed IDH1 mutation and 21.1% of cases showed ATRX loss. Among grade 3 astrocytomas, 66.7% of cases showed IDH1 mutation, and 33.3% of cases showed loss of ATRX. All cases of grade 4 astrocytoma were IDH1-positive, whereas all cases of glioblastoma were IDH1-negative. Among grade 2 and 3 astrocytomas, a few cases showed IDH1 negativity (42.1% and 33.3%, respectively), and a few of them showed retained ATRX (78.9% and 66.7%, respectively). Grade 2 oligodendrogliomas have well-defined cell membranes and clear space around uniform, round, and slightly enlarged nuclei. Glioblastomas displayed negative IDH1 and retained ATRX in all seven cases. Retained ATRX was seen in grade 2 and 3 oligodendrogliomas. Morphology correlated with IHC markers in oligodendrogliomas. The association of both the IHC markers with astrocytomas was not found to be statistically significant (p>0.05). The microscopic and immunohistochemical images of grade 2 astrocytoma and grade 3 oligodendroglioma are depicted in Figure 1 and Figure 2, respectively.

## Discussion

IDH1 mutations in glioblastomas were first observed by Parsons et al. [7]. They observed that IDH1-positive glioblastoma patients have a good prognosis. Improved progression-free survival (PFS) was demonstrated by Sanson et al. [8] in patients with IDH1 mutant GBs. Seven types of IDH1 mutations have been identified in gliomas. The most frequent mutation is R132H, which can be detected by IHC. Remaining mutations are rare and can only be detected by gene sequencing methods. IDH1 and IDH2 mutations are mutually exclusive [6].

IDH1 mutations are typical of lower-grade astrocytomas and oligodendrogliomas and are not found in normal glial tissue or reactive gliosis (IDH-negative) [9]. So, IHC with IDH1 R132H can be used to rule out reactive gliosis. IDH1 mutations were associated with a favorable prognosis than diffuse IDH1 wild-type gliomas [10]. Watanabe et al. [11] observed that there was no case in which IDH mutation occurred after the acquisition of either TP53 mutation or loss of 1p/19q co-deletion, suggesting that IDH1 mutations are very early events in glioma genesis and may affect a common glial precursor. IDH mutations are found in secondary glioblastomas (now called grade 4 astrocytomas), whereas they are absent in primary glioblastomas; thus, it helps in differentiating grade 4 astrocytomas from glioblastoma. Therefore, gene sequencing analysis for IDH1 R132H-negative astrocytic tumors is reserved for grade 4 astrocytoma patients of <55 years of age and not necessary in WHO grade 4 tumors if the age of the patient is >55 years. The frequency of IDH1 mutation in grade 2 astrocytomas varied from 42.5% to 100% in various studies, but in the present study, it was 57.9% (Table 2) [3,4,12-14]. All the grade 4 astrocytomas were IDH1-positive, whereas glioblastomas were negative for IDH1 in the present study, which is similar to the study by Chatterjee et al. [3].

**Table 2:** Frequencies of IDH1 mutation in comparison with other studies IDH1: isocitrate dehydrogenase 1

	Grade 2 astrocytoma	Grade 3 astrocytoma	Grade 4 astrocytoma	Glioblastoma	Grade 2 oligodendroglioma	Grade 3 oligodendroglioma
Present study (n=51) (2023)	57.9%	66.7%	100%	0%	100%	100%
Jain et al. [4] (n=60) (2023)	100%	100%	-	15.62%	83.3%	100%
Sipayya et al. [12] (n=195) (2022)	42.5%	NA	85.7%	1.5%	22.7%	-
Santosh et al. [13] (n=77) (2022)	66.7%	50%	20.7%	-	80.8%	83.3%
Chatterjee et al.[3] (n=80) (2018)	72%	86.7%	100%	0%	-	-
Cai et al. [14] (n=97) (2016)	58%	55.6%	-	15.5%	-	-

ATRX mutation is a feature of astrocytic differentiation, which can be determined by loss of nuclear ATRX. Loss of ATRX has been associated with a better prognosis in gliomas, irrespective of histological grade [15]. According to Wiestler et al. [16], ATRX testing is not only important in the re-classification of mixed oligoastrocytic tumors but also separates a subgroup of IDH mutant astrocytic tumors with a favorable clinical course. In cases of oligoastrocytic tumors, cases showing astrocytic morphology and IDH1 mutant and ATRX retained are more toward oligodendroglial rather than astrocytic tumors, as ATRX and 1p/19q co-deletion are mutually exclusive. In their study, survival analysis divided IDH mutant astrocytic tumors into two groups depending on ATRX status: IDH mutant, ATRX retained, and IDH mutant ATRX lost. Tumors that are IDH mutant and ATRX lost had a significantly better prognosis compared to tumors that are IDH mutant and retained ATRX expression [16]. The frequency of ATRX loss in grade 2 astrocytomas varied from 83.3% to 87% in various studies (Table 3), but in our study, it was less than 21.1% [3,4,13,14]. ATRX was retained in all cases of oligodendrogliomas in the present study, similar to the study by Santosh et al. [13]. ATRX was retained in all cases of glioblastomas in the present study.

**Table 3:** Comparison of frequencies of ATRX loss in various studies ATRX: alpha thalassemia/mental retardation syndrome X-linked

Studies	Grade 2 astrocytoma	Grade 3 astrocytoma	Grade 4 astrocytoma	Glioblastoma	Grade 2 oligodendroglioma	Grade 3 oligodendroglioma
Present study (n=51) (2023)	21.1%	33.3%	55.6%	0%	0%	0%
Jain et al. [4] (n=60) (2023)	83.3%	71.4%	-	3.1%	50%	100%
Santosh et al. [13] (n=77) (2022)	86.7%	75%	86.2%	-	0%	0%
Cai et al. [14] (n=97) (2016)	82%	77.8%	-	12.06%	-	-
Chatterjee et al. [3] (n=80) (2018)	87%	100%	100%	0%	-	-

Various studies have included different IHC and/or molecular markers for the stratification of diffuse gliomas. Cai et al. [14] divided diffuse astrocytomas into three molecular subgroups based on IDH1, ATRX, and Ki67. According to Cai et al., IDH mutant and ATRX-negative tumors have good prognosis, whereas IDH mutant and ATRX retained tumors have intermediate prognosis. This is similar to the molecular classification proposed by Jain et al. [4]. Ki67 values can overlap with grade 2 tumors at one end and grade 4 tumors at the other end [17]. Ki67 values do not appear to be associated with survival in grade 4 IDH mutant tumors [18]. ATRX, 1p/19q co-deletion, and IDH1/IDH2 has been used to create a molecular diagnostic algorithm for diffuse gliomas by Santosh et al. [13]. Since ATRX and 1p/19q co-deletion are mutually exclusive, they proposed that an initial screening test with IDH1 and ATRX is sufficient to identify the majority of diffuse gliomas. 1p/19q co-deletion, which is an expensive test, can be done only in ATRX retained cases. Chatterjee et al. [3] had successfully characterized diffuse gliomas into molecularly defined groups using IDH1 and ATRX in most cases of astrocytomas. Rajeswarie et al. also studied IDH1 and ATRX and concluded that they are highly useful in categorizing diffuse gliomas into histomolecular groups [19].

The limitations of the present study are that usually, if IDH1 is negative in patients aged <55 years by IHC, gene sequencing should be done to rule out other IDH mutations. We have not done gene sequencing in the present study due to financial constraints. 1p/19q deletion by FISH was also not performed to confirm oligodendroglioma. The other limitation is that the sample size is small. A large number of cases should have been studied for the validity of results.

## Conclusions

The frequency of IDH1 mutations in diffuse gliomas was 64.7%. The loss of ATRX in gliomas was less in the present study compared to other studies. Histopathology was well correlated with IHC markers in all cases of oligodendrogliomas and glioblastomas. Morphology along with immunohistochemistry with IDH1 and ATRX can classify gliomas into subtypes without the need for expensive molecular testing in most cases. These surrogate IHC markers are helpful in resource-limited settings where the economic burden on the patients can be minimized. This approach can be adopted as a routine practice in histopathology laboratories for the diagnosis and subtyping of diffuse gliomas. Apart from diagnosis, these markers help to differentiate grade 4 astrocytoma from glioblastoma, which has a poorer prognosis.
